# An immune-related adverse event of Behcet’s-like syndrome following pembrolizumab treatment

**DOI:** 10.1186/s12890-024-02986-y

**Published:** 2024-04-05

**Authors:** Qiao Chen, Deyu Li, Guifeng Zhang, Jiangming Zhong, Li Lin, Zhenhua Liu

**Affiliations:** Department of Medical Oncology, Shengli Clinical Medical College of Fujian Medical University, Fujian Provincial Hospital, Fuzhou, Fujian PR China

**Keywords:** Pembrolizumab, Immune-related adverse event, Immunotherapy, Oral ulcer

## Abstract

**Background:**

In recent years, the emergence of immunotherapy has renewed therapeutic modality. Different from traditional anti-tumor therapy, immune-related adverse events of skin, gastrointestinal tract, liver, lung, endocrine glands commonly occurred. At present, only one case of immune-related adverse event of Behcet’s-like syndrome following pembrolizumab treatment was reported in USA, and no one is reported in China.

**Case presentation:**

Here, we report a rare case of Behcet’s-like symptom following pembrolizumab treatment. A 43-year-old female was diagnosed as lymph node and bone metastasis of adenocarcinoma with unknown primary lesion, probably being of pulmonary origin. She was treated with pembrolizumab 200 mg every three weeks in combination with chemotherapy for 6 cycles, followed by pembrolizumab monotherapy maintenance. However, she developed Behcet’s-like syndrome with oral ulcer, genital uler, phlebitis, and vision loss after 9 cycles of pembrolizumab treatment. She was treated with prednisone 5 mg orally three times a day. Two weeks later, dose of glucocorticoid gaven to the patient gradually decreased with improved symptoms. After a treatment-free withdrawal period, the patient requested to continue pembrolizumab treatment. Unfortunately, the above symptoms recurred on the second day following pembrolizumab treatment, and glucocorticoid was taken once again. The symptoms improved and the condition was under control.

**Conclusions:**

In view of the exponential growth of immunocheckpoint inhibitors (ICIs) in a variety of tumors, we should be alert to related adverse events, especially the rare rheumatic manifestations.

## Background

In recent years, the emergence, development and rapid uptake of checkpoint inhibitors, a modern form of immunotherapy, resulted in changes to the way numerous cancers are managed. Different from traditional anti-tumor therapy, the function mechanism of immune checkpoint inhibitors‘(ICIs) is unique, and their adverse events have also formed a unique disease spectrum, known as immune-related adverse events (irAEs)[1, 2]. IrAEs may occur on multiple organs and systems including the skin, gastrointestinal tract, liver, lung, endocrine glands, and ICIs could cause rheumatic manifestations, such as inflammatory arthritis, rheumatic myalgia, myositis, vasculitis, Sjogen syndrome and systemic lupus erythematosus. Presently, only one case of immune-related adverse event of Behcet’s-like syndrome following pembrolizumab treatment was reported in USA, and no one is reported in China. Here, we would like to share a rare case of Behcet’s-like syndrome with oral ulcer, genital ulcer, phlebitis, and vision loss adverse events following pembrolizumab treatment.

## Case presentation

**Fig. 1:**
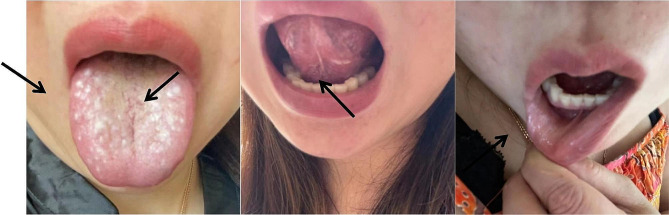
Clinical manifestations before treatment(oral ulcers located on the superior surface of tongue, the inferior surface of tongue and surface of lips)

A 43-year- old female patient with a 3 cm × 4 cm mass on the left neck was examined in November 2021. She had a slightly elevated levels of tumor markers of carcinoembryonic antigen(CEA), Carbohydrate antigen 199(CA199) and carbohydrate antigen 125(CA125). Whole-body Positron Emission Tomography-Computed Tomography(PET/CT) revealed the multiple small nodules in the left lung base, and multiple bone and lymph node metastasis. The case was clinically diagnosed as adenocarcinoma, probably being of pulmonary origin via fine-needle aspiration smear. Immunohistochemistry: CK7 (+), CK20 (-), TTF-1 (+), Nap (+), PAx8 (-), P63 (-), P40 (-), Syn (-), CDX2-88 (-), CD68/kpl (-), PD-LI 22C3 (TPS:85%). Gene detection: echinoderm microtubule-associated protein-like 4(EML4)-anaplastic lymphoma kinase(ALK) fusion (5.7%), microsatellite stability(MSS). Finally, the case was pathologically diagnosed as lymph node and bone metastasis of adenocarcinoma with unknown primary focus, probably being of pulmonary origin. From December 2021 to April 2022, she received the first-line treatment of pembrolizumab 200 mg and bevacizumab (used in the first cycle, and then stopped due to nosebleed) combined with systemic chemotherapy of pemetrexed 500 mg/m2 and cisplatin 75 mg/m2 every three weeks for 6 cycles. The patient’s condition improved based on CT examination. According to Response Evaluation Criteria In Solid Tumors(RECIST) standard, the curative effect of tumor was partial response. Then pembrolizumab 200 mg was given regularly every 3 weeks for maintenance treatment. The third cycle of maintenance treatment with 200 mg pembrolizumab was given in June 2022. On the second day after treatment, many rice-grain-size oral ulcers appeared. Originally, oral ulcers were located on the inferior surface of tongue, and then gradually expanded to the superior surface of tongue, surface of lips, gingiva, pharynx, hard palate (Fig. 1), and spreading on a large scale with slight pain. Taking vitamin C failed to provide relief from orall. Meanwhile, two ulcers in the perineum and three perianal ulcers appeared with thumb size and occasional pain. Thusly, the patient went to the gynecological department for treatment, however, monilial vaginitis could not be excluded, and low curative effect was observed of clotimazole cream.During the onset of the disease, she did not have gastrointestinal symptoms such as nausea, vomiting, abdominal pain, bloating, diarrhea, etc. But she appeared pain in the veins on the back of her hand, accompanied by blurred vision.She did not go to see an ophthalmologist or undergo relevant vascular examinations, and polyethylene glycol eye drops was given to her eyes. The symptoms failed to be improved significantly, and it was not effective after medicine for external use or oral Chinese medicine. Both the autoimmunity test and ANCA test are negative. The ulcer was aggravated and expanded, accompanied with blurred vision and phlebitis. She went to a rheumatologist for help in July 2022, and was considered Behcet’s-like disease.She was not tested for HLA typing. Then, she was treated with prednisone 5 mg orally three times a day, and her symptoms improved. Two weeks later, many ulcers in the whole body basically healed, and vision returned to normal level. Subsequently, the dose of prednisone was gradually reduced to discontinuation. After a treatment-free withdrawal period, the fourth cycle of maintenance treatment with 200 mg pembrolizumab was given at the request of the patient in August 2022. On the second day of pembrolizumab treatment, the above symptoms with multiple oral ulcers and perineal ulcers recurred, and immediately, the patient was given prednisone orally, and the symptoms were soon controlled.

## Discussion and conclusions

First-line therapy for advanced non-small-cell lung cancer that lacks targetable mutations is platinum-based chemotherapy. Notably, immunotherapies have raised hope for a successful control of metastatic lung adenocarcinoma. Pembrolizumab, a humanized monoclonal antibody against programmed death 1 (PD-1), has resulted in remarkably longer survival and fewer adverse events. Pembrolizumab could relieve immunosuppression mediated by PD-1 pathway, and enhance its ability to kill tumor cells by activating T cell function [3–5]. However, the activated T cells will attack normal tissue, and produce crossed immune reaction which induces autoimmune inflammation. It produces irAEs similar to autoimmune diseases [6]. Although rheumatic irAE is not a common irAE, it has been paid more and more attention with the increase of immunotherapy in clinical use in recent years. For example, a large tertiary cancer center in Israel found that 14 (3.5%) of 400 patients had rheumatic irAE [7]. The incidence of rheumatic irAE is related to several factors, such as the type and dose of ICI, the combination of ICI used. Compared with patients treated with PD-1/PD-L1 inhibitors, patients treated with CTLA-4 inhibitors are more likely to have irAE [8]. In patients with pre-existing rheumatologic conditions, incluing Behcet’s syndrome, There is a risk of flare with combination ipilimumab and anti-PD1. Close monitoring is essential [9]. A study conducted by Cardona AF et al. showed that Oral ulcers which were insensitive to steroid treatment associated with immune checkpoint inhibitors might be related to both Behçet’s disease and graft versus host disease physiopathologically [10]. The use of anti PD-1 drugs will alter the immune system and cause cross-reaction with several antigens presenting in oral epithelium. It is reported that the changes of oral mucosa related to anti-PD-1 drugs are about 1% for Nivolumab and 2% for Pembrolizumab, beyond that no serious event was observed [11]. The incidence of grade 1 and grade 2 mucositis with Nivolumab and Pembrolizumab ranges from 1 to 2%. Oral mucositis caused by PD-1 seemed to be more common than CTLA-4 inhibitors, which are rare in immunotherapy in general [12].

A retrospective study showed that 42.5% of patients with grade 2 or above IrAEs who had been treated with ICIs had recurrence of the original irAEs again [13]. The first case of Behcet’s like syndrome following pembrolizumab treatment was reported that an elderly male with facial metastatic melanoma received 24 months on pembrolizumab therapy. Then he developed Behcet’s like syndrome with corneal ulcers, oral and genital ulcers.His symptoms improved after treatment with prednisone and colchicine [14]. This case we shared may be the second case of Behcet’s like syndrome associated with irAE of pembrolizumab treatment. And it is the first time to report the Behcet’s-like syndrome with oral ulcer, genital uler, phlebitis, and vision loss after pembrolimab treatment in China.

There is a certain correlation between Behcet’s syndrome and myelodysplastic-syndromes(MDS). Some patients with MDS may experience Behcet’s syndrome. It is more common with intestinal involvement and often accompanied by fever, especially with trisomy 8. However, the reason for the association between them is not clear, whether it is the autoimmune background causing MDS, or Behcet’s syndrome is the paraneoplastic state of MDS [15].

Behcet’s syndrome is an autoimmune disease based on vasculitis with diverse clinical manifestations. Due to the lack of specific experimental indicators so far, Behcet’s syndrome is easily misdiagnosed or undiagnosed. The main manifestations of Behcet’s syndrome are recurrent oral ulcer (at least three times in one year), genital ulcer, eye damage, skin damage. Besides, Behcet’s syndrome can also attack the gastrointestinal tract, heart, and peripheral blood vessels [16], its onset age was mostly 20–40 years old. The exact pathogenesis of Behcet’s Disease (BD) is still unclear with certain genetic susceptibility. Research shows that infection factors may play a role in triggering and/or continuing BD. The production or increased reactivity of pro-inflammatory components of innate immune response (TLR, neutrophils, NK cells) to these triggers may be a key step in the pathogenesis of BD. Inflammation has been comfirmed to cause a typical Behcet’s manifestations, including orogenic ulcer, uveitis, and skin damage [17]. The International Criteria for BD (ICBD) in 2014 (Table 1) is used to establish classification criteria of BD. It shows a total score of 4 points or more can be diagnosed as Behcet’s disease [18].The symptoms of this case are very similar to Behcet’s disease, but it is not enough to diagnose Behcet’s disease at present.It requires further observation and follow-up. Glucocorticoids are given to suppress immunity, reduce inflammation and relieve symptoms.

**Table 1:** The international criteria for Behcet’s disease

Symptom	Score*
Ocular lesions	Two points
Genital aphthosis	Two points
Oral aphthosis	Two points
Skin lesions	One point
Neurological manifestations	One point
Vascular lesions	One point
Pathergy	One point

*: Four or more points satisfy criteria for Behcet’s disease.

Approximately 10% of cancer patients receiving ICIs treatment present rheumatic and musculoskeletal irAEs [19]. According to the type and severity of adverse reactions in rheumatism, different treatment methods are adopted and different doses of corticosteroids are used. For moderate or severe cases, it is recommended to stop ICI treatment and refer to a rheumatologist. High-dose corticosteroids, intravenous immunoglobulin, human interleukin 6 receptor inhibitor, tumor necrosis factor-α inhibitor and/or plasma exchange may be considered if necessary [20].

At present, although exponentially increasing ICIs are used in malignant melanoma, lymphoma, renal cancer, lung cancer and other cancers, irAEs can not be ignored, especially the rare rheumatic manifestations. We should improve the ability to recognize the irAEs as early as possible.

## Data Availability

The data that support this case report are available from the corresponding author on reasonable request.

## Abbreviations

ICIs: Immune checkpoint inhibitors
irAEs: Immune-related adverse events
PD-1: Programmed death 1
BD: Behcet’s Disease
ICBD: The International Criteria for BD
CEA: Carcinoembryonic antigen
CA199: Carbohydrate antigen 199
CA125: Carbohydrate antigen 125
EML4: Echinoderm microtubule-associated protein-like 4
ALK: Anaplastic lymphoma kinase
IHC: Immunohistochemistry
RECIST: Response Evaluation Criteria In Solid Tumors
MSS: Microsatellite stability
PET/CT: Positron Emission Tomography-Computed Tomography
MDS: Myelodysplastic-syndromes
